# Association between sarcopenia and kidney stones in United States adult population between 2011 and 2018

**DOI:** 10.3389/fnut.2023.1123588

**Published:** 2023-03-06

**Authors:** Yifan Zhang, Changxiu Tian, Yidi Wang, Houliang Zhang, Jinliang Ni, Wei Song, Huajuan Shi, Tao Zhang, Changbao Xu, Keyi Wang, Bo Peng

**Affiliations:** ^1^Department of Urology, Shanghai Tenth People’s Hospital, School of Medicine, Tongji University, Shanghai, China; ^2^Department of Urology, Shanghai Putuo District People’s Hospital, Tongji University, Shanghai, China; ^3^Department of Urology, Second Affiliated Hospital of Zhengzhou University, Zhengzhou, China

**Keywords:** sarcopenia, sarcopenia index, kidney stones, cross-section study, NHANES

## Abstract

**Purpose:**

To investigate the relationship between kidney stones and sarcopenia in United States adult population between 2011 and 2018.

**Materials and methods:**

We conducted a cross-section study based on the National Health and Nutrition Examination Survey (NHANES) including 39,156 individuals. Sarcopenia was assessed by the sarcopenia index. Association between kidney stones and sarcopenia verified by multiple logistic regression analysis and dose–response curves analysis using restricted cubic spline (RCS) regression. Meanwhile, propensity score matching (PSM) was performed to exclude the effect of confounding variables.

**Results:**

There were 9,472 participants in the study by our accurate enrollment screening process. The odds of kidney stones decreased significantly with the increase of sarcopenia index. Logistic regression analysis showed that sarcopenia expressed significant differences in the participants which suffered kidney stone before PSM (*p* < 0.001). In model 4, adjusting all relevant covariates shown that adjusted odds ratio (aOR) of the 95% confidence intervals for kidney stones in all participants, age <39 years and age ≥40 years, were, respectively, 1.286 (1.006–1,643), 1.697 (1.065–2.702), and 0.965 (0.700–1.330) for sarcopenia, and *p* values were 0.044, 0.026, and 0.827. After performing PSM, the aOR of the 95% in modal 4 for kidney stones in all participants and age <40 year were 2.365 (1.598–3.500) and 6.793 (2.619–17.6180), respectively (*p* < 0.01), and especially the aOR in participants (age ≥40) was 1.771(1.138–2.757) with *p* value being 0.011.

**Conclusion:**

Sarcopenia was positively related to the potential risk of kidney stones in the United States adult population.

## Introduction

Urolithiasis was the most known urology disease around the worlds, and it caused a certain problem of various groups of patients. Kidney stones as the most common type of urolithiasis had an increasing prevalence over the past decades, placing high costs and clinical burdens on the healthcare system (1). In the United States, kidney stones spent more than $2.1 billion on healthcare in 2000 (2). Kidney stones formation had been shown to be related to environmental and genetic factors such as climate, diet, fluid intake, smoking, caffeine, age, gender, body mass index (BMI), and type 2 diabetes (DM) (3).

Sarcopenia had been defined as a progressive and systemic skeletal muscle disease associated with accelerated loss of muscle mass and function. More recently, sarcopenia had been defined as a disease with many adverse effects such as falls, functional decline, weakness, and death (4). In 2018, EWGSOP2 updated the definition and diagnostic guidelines for sarcopenia, stating that people with low muscle strength, muscle mass/mass, and physical performance would be diagnosed with sarcopenia (5). Interestingly, we found a strong correlation between sarcopenia and stones, and it had not been reported in any study.

The purpose of this study was to investigate the exposure-response relationship between sarcopenia and the incidence of kidney stones in the National Health and Nutrition Examination Survey (NHANES) from 2011 to 2018.

## Materials and methods

### Study design and participants

**Figure 1:**
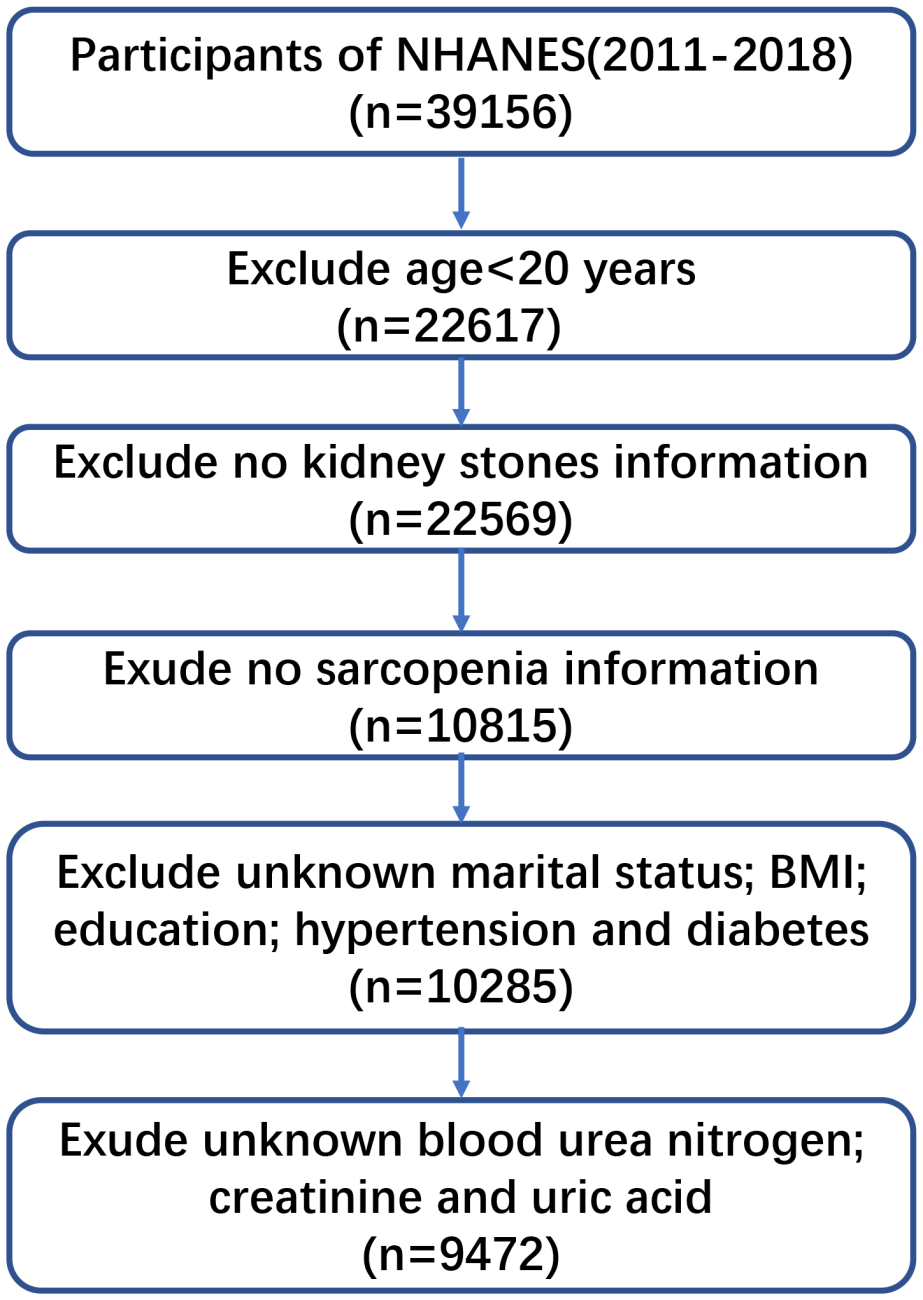
Schematic flow diagram of inclusion and exclusion criteria for our study cohort.

NHANES was a program that assessed the health and nutritional status of the American people, which combined interviews and physical examinations to collect data focusing on diet and health-related incidents. The study protocol was endorsed by the NHANES Institutional Review Board, and all participants provided informed consent during the survey. In this study, there were 39,156 participants in eight NHANES cycles 2011–2012, 2012–2013, 2013–2014, 2014–2015, 2015–2016, 2016–2017, and 2017–2018. The study excluded participants if their conditions met any of the following: (1) Their age was under 20 years old (*n* = 22,617); (2) They had no kidney stones or sarcopenia information (*n* = 10,815); (3) Their marital status, BMI, education, hypertension, and diabetes were unknown (*n* = 10,285); (4) Their blood urea nitrogen, creatinine and uric acid were unknown (*n* = 9,472). Finally, we collected 9,472 participants, as shown in Figure 1.

### Exposure variable and outcomes variable

Sarcopenia was the major exposure variable in this study. Sarcopenia was assessed by the sum of the muscle mass of the four limbs (ALM, appendicular lean mass). Dual-energy X-ray absorptiometry (DEXA) was used to measure ALM by NHANES. Pregnant participants and those participants who weight more than 136.4 kg or height more than 192.5 cm were excluded from the study, because these individuals could not be measured by DEXA. We calculated the sarcopenia index as following: sarcopenia index = total appendicular skeletal muscle mass (in kg)/BMI (kg/m^2^). Sarcopenia was defined by sarcopenia index: it judged to exist sarcopenia if sarcopenia index of men and women was less than 0.789 and 0.512, respectively.

The major outcome variable of the study was the kidney stones history. The kidney stones history was assessed by the answers of participants. The participants who had suffered kidney stones were divided into kidney stones groups, and the rest of participants were divided into non-kidney stones groups.

### Potential covariates

Based on previous studies, relevant covariates were identified. Continuous variables included age (<40 years/≥40 years); body mass index (BMI); blood urea nitrogen; and creatinine and uric acid. Categorical variables included Gender (male/female); Race (non-Hispanic white/non-Hispanic black/Mexican American/other Hispanic/other); Education level (less than high school/high school or equivalent/college or above); Marital status (married/unmarried); Hypertension; Smoking status (never/former/current); Alcohol use; Vigorous recreational activities; Moderate recreational activities; Sarcopenia; blood urea nitrogen; creatinine; and uric acid.

### Statistical analysis

Continuous variables and categorical variables were presented as mean ± standard deviation and number (percentage), respectively. Comparisons among different groups were performed by *t*-tests and one-way ANOVA tests for normally distributed continuous variables, then non-normal continuous variables was compared by independent-samples Kruskal–Wallis tests, and categorical variables among different groups were determined statistical differences by Chi-square tests. Logistic regression analyzed the relationship between sarcopenia and the presence of kidney stones, using the corrected odds ratio (OR) and corresponding 95% confidence intervals (CI) to describe the associations. In the extended model, model 1 was univariate analysis; model 2 was modified gender, age, and race; model 3 was model 2 plus education level, marital status, and BMI; model 4 was model 3 adding hypertension, smoking status, alcohol use, physical activities, blood urea nitrogen, creatinine, and uric acid.

The powerful tool of restricted cubic spline (RCS) function was applied to describing dose–response relationships between continuous variables and outcomes, and was also utilized in our research to characterize the dose–response relationship among sarcopenia index and kidney stone risk, adjusted for model variables. All statistical analyses were conducted using IBM SPSS 20.0 software (IBM, United States) and GraphPad Prism8 software (GraphPad Software Inc., La Jolla, CA, United States). *p*-Values less than 0.05 were considered to be statistically significant.

## Results

### Participants’ characteristics

As shown in Figure 1, 39,156 participants were recruited from the NHANES (2011–2018). During screening, basic characteristics of participants are summarized in Table 1. Eight thousand seven hundred and thirteen (92%) participants were divided into none-stone formers group and 759 (8%) into stone formers. Gender and education level had no significance between the two groups (*p* > 0.05). The others had significant differences between both groups, including age, race, marital status, BMI, hypertension, smoking status, alcohol use, vigorous recreational activities, moderate recreational activities, sarcopenia, blood urea nitrogen, creatinine, and uric acid (*p* < 0.05). Supplementary Table S1 summarizes basic characteristics of participant with no-sarcopenia group and sarcopenia group. Eight thousand six hundred and sixty-one (91.4%) participants were divided into no-sarcopenia and 811 (8.6%) into sarcopenia. We found that people with sarcopenia were more likely to be elderly (age ≥ 40 years), married, Mexican American, college degree or above, higher BMI, less vigorous recreational activities, less moderate recreational activities, lower creatinine, and higher uric acid (*p* < 0.05).

**Table 1:** Baseline characteristics of participants between 2011 and 2018.

Characteristic	Total	None-stone formers	Stone formers	*p*-Value
	*N* (%)	*N* (%)	*N* (%)	
Total patients	9,472	8,713 (92.0)	759 (8.0)	
Gender				0.587
Male	4,657 (49.2)	4,291 (49.2)	366 (48.2)	
Female	4,815 (50.8)	4,422 (50.8)	393 (51.8)	
Age				<0.001
<40 years	4,793 (50.6)	4,518 (51.9)	275 (36.2)	
≥40 years	4,679 (49.4)	4,195 (48.1)	484 (63.8)	
Race				<0.001
Non-Hispanic white	3,428 (36.2)	3,055 (35.1)	373 (49.1)	
Non-Hispanic black	1934 (20.4)	1837 (21.1)	97 (12.8)	
Mexican American	1,374 (14.5)	1,274 (14.6)	100 (13.2)	
Other Hispanic	948 (10.0)	856 (9.8)	92 (12.1)	
Other	1788 (18.9)	1,691 (19.4)	97 (12.8)	
Education level				0.695
Less than high school	1,610 (17.0)	1,479 (17.0)	131 (17.3)	
High school or equivalent	2063 (21.8)	1907 (21.9)	156 (20.6)	
College or above	5,799 (61.2)	5,327 (61.1)	472 (62.2)	
Marital status				0.011
Married	4,625 (48.8)	4,221 (48.4)	404 (53.2)	
Unmarried	4,847 (51.2)	4,492 (51.6)	355 (46.8)	
BMI (kg/m^2^)				<0.001
<25.0	3,001 (31.7)	2,835 (32.6)	166 (21.9)	
25.0–29.9	2,966 (31.3)	2,731 (31.4)	235 (31.0)	
≥30.0	3,497 (37.0)	3,139 (36.1)	358 (47.2)	
Hypertension				<0.001
Yes	2,223 (23.5)	1945 (22.3)	278 (36.6)	
No	7,249 (76.5)	6,768 (77.7)	481 (63.4)	
Smoking status				<0.001
Never	5,733 (60.5)	5,332 (61.2)	401 (52.8)	
Former	1,613 (17.0)	1,457 (16.7)	156 (20.6)	
Current	2,126 (22.4)	1924 (22.1)	202 (26.6)	
Alcohol use				0.010
Yes	7,040 (74.3)	6,446 (74.0)	594 (78.3)	
No/Unknown	2,432 (25.7)	2,267 (26.0)	165 (21.7)	
Vigorous recreational activities				<0.001
Yes	2,967 (31.3)	2,779 (31.9)	188 (24.8)	
No	6,505 (68/7)	5,934 (68.1)	571 (75.2)	
Moderate recreational activities				0.042
Yes	4,277 (45.2)	3,961 (45.5)	316 (41.6)	
No	5,195 (54.8)	4,752 (54.5)	443 (58.4)	
Sarcopenia				<0.001
Yes	811 (8.6)	715 (8.2)	96 (12.6)	
No	8,661 (91.4)	7,998 (91.8)	663 (87.4)	
Blood urea nitrogen (mg/dl)	12.53 ± 4.50	12.48 ± 4.40	13.16 ± 5.42	<0.001
Creatinine (mg/dl)	0.85 ± 0.37	0.85 ± 0.34	0.89 ± 0.58	<0.001
Uric acid (mg/dl)	5.32 ± 1.39	5.32 ± 1.39	5.35 ± 1.39	<0.001

For categorical variables, *p* values were analyzed by Chi-square tests. For continuous variables, the *t*-test was used. BMI, body mass index.

### Sarcopenia and kidney stones

**Figure 2:**
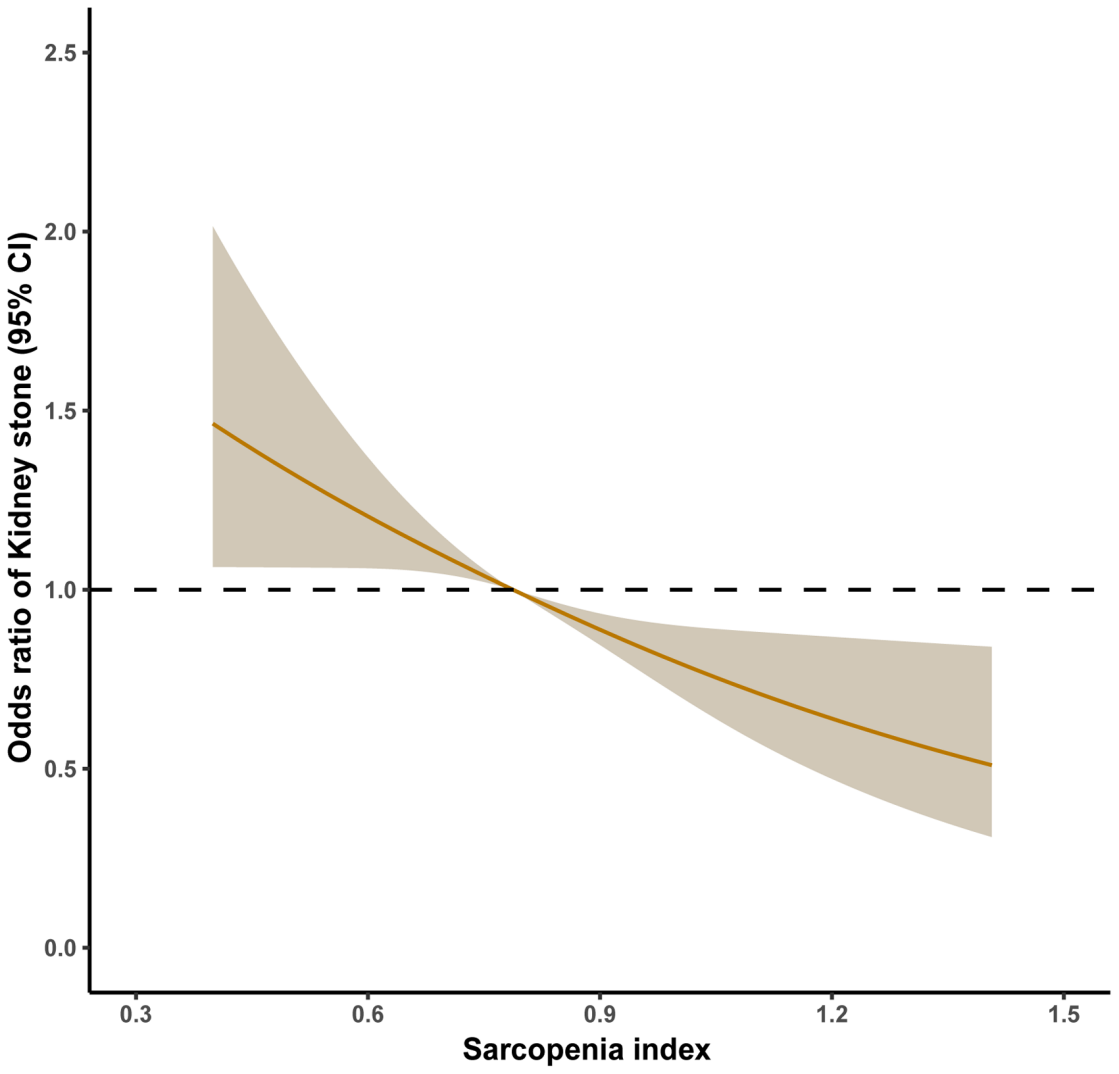
Relative risk for kidney stones based on sarcopenia index before PSM. The shaded areas represent upper and lower 95% CIs. Adjustment factors are as same as which presented in extended model 4. Restricted cubic spline (RCS) plot of the association between sarcopenia index and kidney stones. The solid and dashed lines represent the odds ratios and 95% confidence intervals.

Figure 2 shows the dose–response relationships between sarcopenia index and kidney stones. There was a no-linear association between LMA (lumbar muscle area) and the prevalence of kidney stones. It showed that the prevalence of kidney stones decreased with increase of sarcopenia index. Then, logistic regression analysis further confirmed that sarcopenia was positively related with prevalence of kidney stones (Table 2). The adjusted odds ratio (aOR) of all participants was 1.620 (95% CI, 1.290–2.033), 1.458 (95% CI, 1.152–1.846), 1.287 (95% CI, 1.010–1.639), and 1.286 (95% CI, 1.006–1.643), respectively (*p* < 0.05). The adjusted odds ratio (aOR) of participants (<40 years) was 1.955 (1.304–2.930), 2.089 (1.377–3.169), 1.926 (1.254–2.959), and 1.697 (1.065–2.702), respectively (*p* < 0.05). However, there were no significant differences about aOR on participants (≥40 years). There were adjusted covariate in four models: model 1: univariate analysis; model 2: gender, age and race; model 3: model 2 plus education level, marital status, and BMI; and model 4: model 3 plus hypertension, smoking status, alcohol use, physical activities, blood urea nitrogen, creatinine, and uric acid.

**Table 2:** Logistic regression analyzed the relationship between sarcopenia and the presence of kidney stones.

	Model 1		Model 2		Model 3		Model 4	
	aOR (95% CI)	*p*	aOR (95% CI)	*p*	aOR (95% CI)	*p*	aOR (95% CI)	*p*
**All participants**								
*Sarcopenia*								
Yes	1.620 (1.290–2.033)		1.458 (1.152–1.846)		1.287 (1.010–1.639)		1.286 (1.006–1.643)	
No	Reference		Reference		Reference		Reference	
*p* for trend	<0.001		0.002		0.041		0.044	
**Age <40 years**								
*Sarcopenia*								
Yes	1.955 (1.304–2.930)		2.089 (1.377–3.169)		1.926 (1.254–2.959)		1.697 (1.065–2.702)	
No	Reference		Reference		Reference		Reference	
*p* for trend	0.001		0.001		0.003		0.026	
**Age ≥40 years**								
*Sarcopenia*								
Yes	1.311 (0.995–1.727)		1.270 (0.955–1.687)		1.085 (0.810–1.454)		0.965 (0.700–1.330)	
No	Reference		Reference		Reference		Reference	
*p* for trend	0.054		0.100		0.584		0.827	

Adjusted covariates: model 1: univariate analysis; model 2: gender, age and race; model 3: model 2 plus education level, marital status, and BMI; model 4: model 3 plus hypertension, smoking status, alcohol use, physical activities, blood urea nitrogen, creatinine and uric acid. BMI, body mass index; CI, confidence interval; aOR, adjusted odds ratio.

### Association after propensity score matching

**Figure 3:**
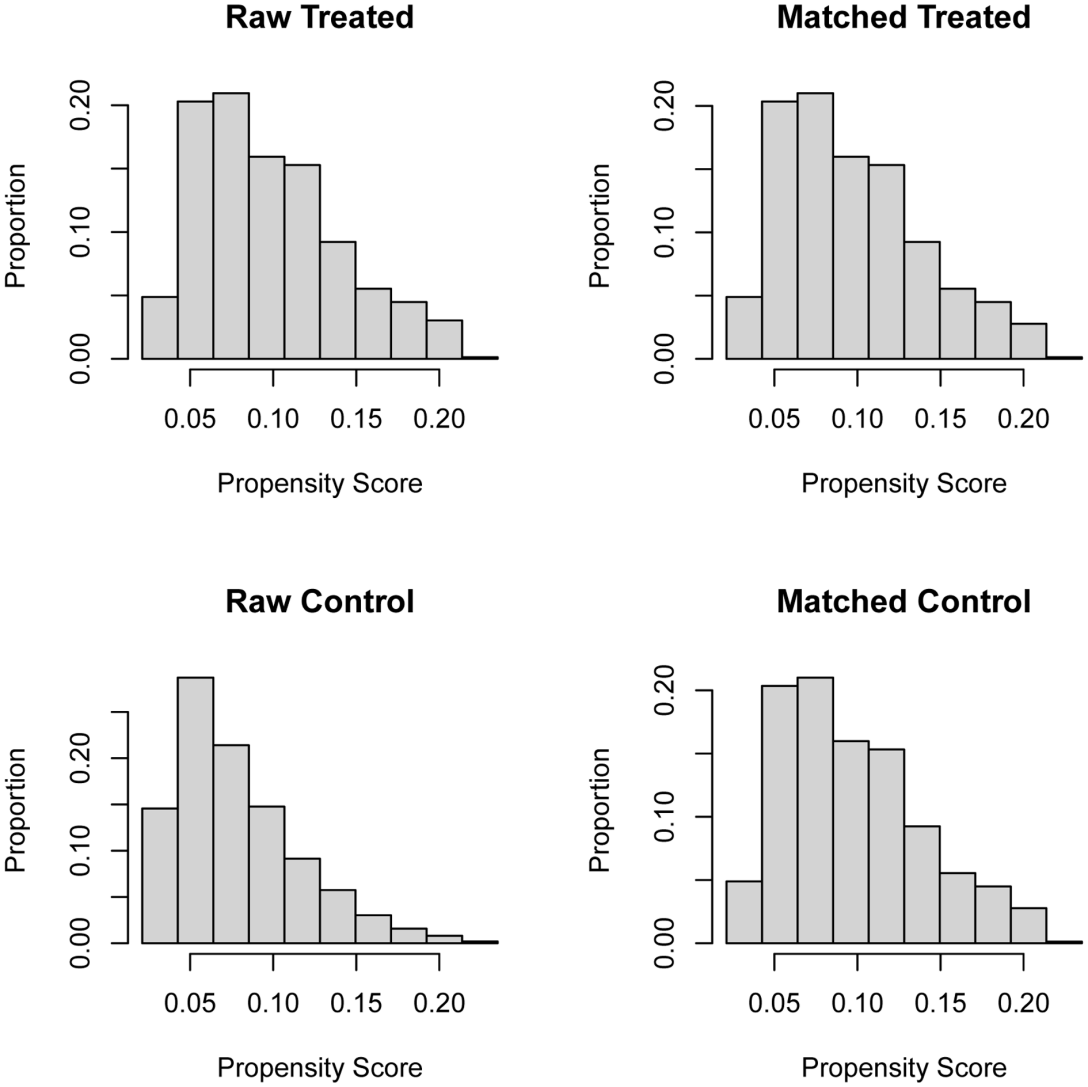
Distribution of propensity score before and after matching.

**Figure 4:**
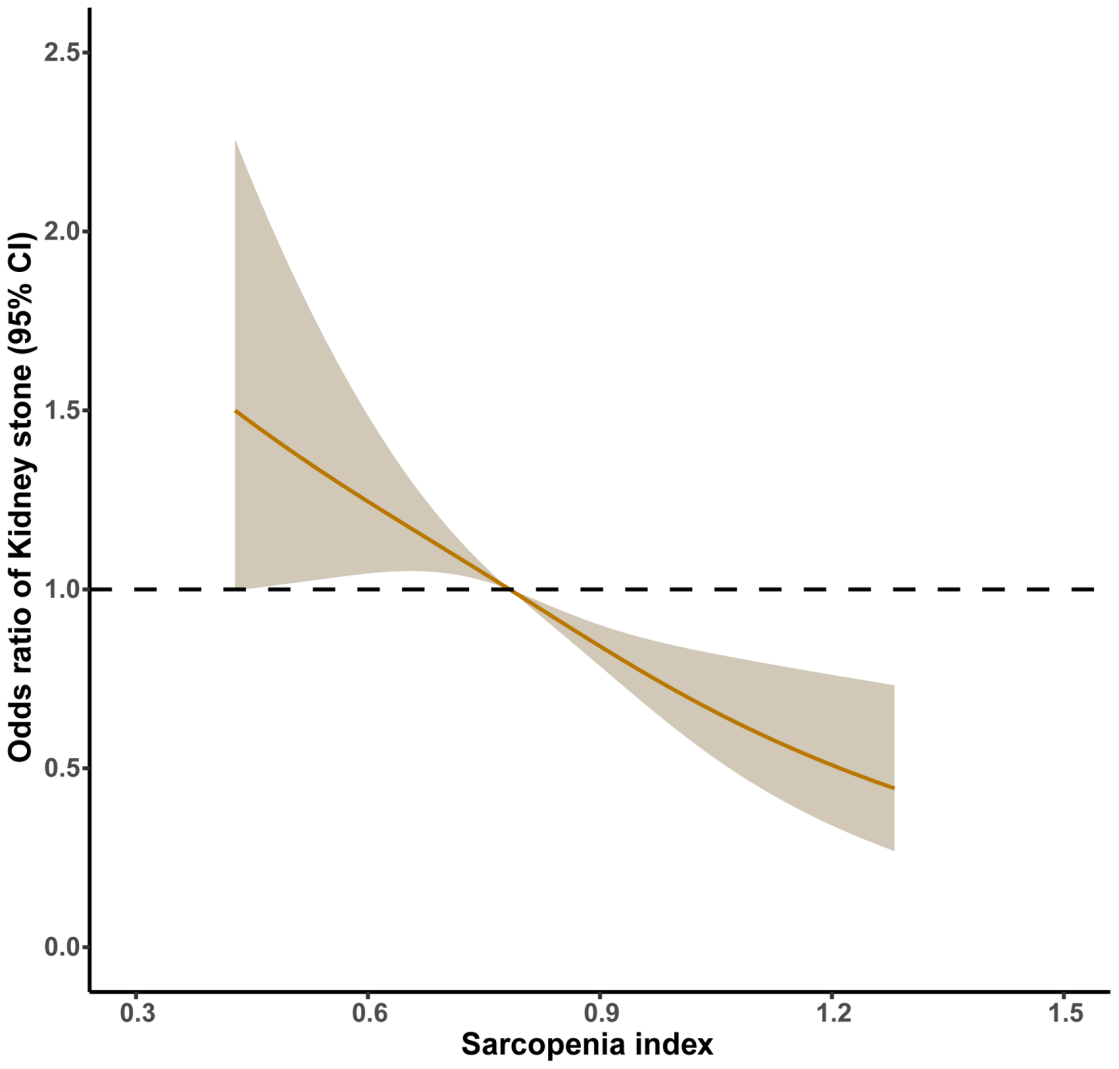
Relative risk for kidney stones based on sarcopenia index after PSM. The shaded areas represent upper and lower 95% CIs. Adjustment factors are as same as which presented in extended model 4. Restricted cubic spline (RCS) plot of the association between sarcopenia index and kidney stones. The solid and dashed lines represent the odds ratios and 95% confidence intervals.

Participants in the study were performed propensity score matching, because of differences between none-stone formers group and formers group. The results after propensity score matching are shown in Figures 3, 4. After propensity score matching, logistic regression analysis clearly shown that sarcopenia was positively related with prevalence of kidney stones. Table 3 displays that *p*-values were less than 0.05 among participants (all, <40 years and ≥40 years) in four models. The adjusted odds ratio (aOR) of all participants was 2.325 (1.602–3.376), 2.300 (1.572–3.366), 2.342 (1.588–3.455), and 2.365 (1.598–3.500), respectively (*p* < 0.01). Then, the adjusted odds ratio (aOR) of participants (<40 years) was 5.600 (2.287–13.710), 5.945 (2.362–14.964), 6.334 (2.479–16.180), and 6.793 (2.619–17.618), respectively (*p* < 0.01). Finally, the adjusted odds ratio (aOR) of participants (≥40 years) was 1.787 (1.173–2.723), 1.761 (1.148–2.700), 1.756 (1.132–2.723), and 1.771 (1.138–2.757), respectively (*p* < 0.05). There were adjusted covariates in four models: model 1: univariate analysis; model 2: gender, age, and race; model 3: model 1 plus education level, marital status, and BMI; and model 4: model 3 plus hypertension, smoking status, alcohol use, physical activities, blood urea nitrogen, creatinine, and uric acid.

**Table 3:** Logistic regression analyzed the relationship between sarcopenia and the presence of kidney stones.

	Model 1	Model 2	Model 3	Model 4
	aOR (95% CI)	aOR (95% CI)	aOR (95% CI)	aOR (95% CI)
**All participants**				
*Sarcopenia*				
Yes	2.325 (1.602–3.376)	2.300 (1.572–3.366)	2.342 (1.588–3.455)	2.365 (1.598–3.500)
No	Reference	Reference	Reference	Reference
*p* for trend	<0.001	<0.001	<0.001	<0.001
**Age <40 years**				
*Sarcopenia*				
Yes	5.600 (2.287–13.710)	5.945 (2.362–14.964)	6.334 (2.479–16.180)	6.793 (2.619–17.618)
No	Reference	Reference	Reference	Reference
*p* for trend	<0.001	<0.001	<0.001	<0.001
**Age ≥40 years**				
*Sarcopenia*				
Yes	1.787 (1.173–2.723)	1.761 (1.148–2.700)	1.756 (1.132–2.723)	1.771 (1.138–2.757)
No	Reference	Reference	Reference	Reference
*p* for trend	0.007	0.010	0.012	0.011

Adjusted covariates: model 1: univariate analysis; model 2: gender, age and race; model 3: model 2 plus education level, marital status, and BMI; model 4: model 3 plus hypertension, smoking status, alcohol use, physical activities, blood urea nitrogen, creatinine, and uric acid. BMI, body mass index; aOR, adjusted odds ratio.

Figure 4 shows the dose–response relationships between sarcopenia index and kidney stones after propensity score matching. There was a no-linear association between sarcopenia index and the prevalence of kidney stones. Respectively comparing Figures 4, 2, Table 2, 3, it was proved that sarcopenia had more dramatic impact on the prevalence of kidney stones especially after PSM. With increasing of sarcopenia index, participants had lower prevalence of kidney stones.

## Discussion

Kidney stones caused a series of damages to patient health and brought great social, economic, and healthy burden, due to its high incidence and recurrence rate. Therefore, it was essential for us to discover the risk factors of kidney stones, and the risk factors might help us to decrease the recurrence of stone disease and reduce burden of society and medicine. Our study discussed the relationship between sarcopenia and kidney stones. Firstly, we explored the association between sarcopenia and kidney stones. Then, we concluded that the odds of kidney stones decreased significantly with the increase of sarcopenia index which was negatively related to sarcopenia, and it was proved by the dose–response curve. However, we found that sarcopenia was not independent risk factor of kidney stones in participants (≥40 years). Therefore, PSM was performed to exclude the effects of other variables. After performing PSM, all participants showed that sarcopenia had the positively impact on prevalence of kidney stones, and the aOR in participants (age ≥40) in model 4 was 1.771 (1.138–2.757). In conclusion, sarcopenia was the independent hazard factor of kidney stones.

Risk factors of kidney stones had increased around the world, and it might influence the more diagnosis of kidney stone. The formation of kidney stones was closely related to risk factors such as age, gender, dietary structure, environmental factors, genetics, abnormal urinary anatomy, and infection (3). Especially, higher rates of obesity; diabetes; more intake of salt and animal protein; higher consumption of sugary beverages; global warming; less exercise; and sedentary behavior might contribute to higher incidence and prevalence of kidney stones (6–8). In recent years, studies had found a positive correlation between BMI, waist circumference, and the risk of kidney stones (9). In general, as BMI increases, so does visceral obesity and hepatic steatosis, both of which were associated with low levels of urinary PH, and low urinary PH could lead to the development of uric acid stones (10). Adipose tissue, as an endocrine organ, was a source of adipokines and inflammatory cytokines that can lead to insulin resistance, inflammatory, and enhanced oxidative stress states, and these conditions would lead to stone crystal formation (11, 12). Sarcopenia was defined as a progressive and systemic skeletal muscle disease involving an accelerated loss of muscle mass and function, which was associated with increased adverse outcomes, including falls, functional decline, weakness, and death (13). Age-related mechanisms that promoted sarcopenic episodes including inflammation, immune aging, anabolic resistance, and increased oxidative stress (14). We found that the pathophysiological mechanisms of sarcopenia and kidney stones shared some of the same risk factors, such as obesity, insulin resistance, lack of physical activity, and chronic inflammation etc. In obese patients, ectopic deposition of fat in the liver and skeletal muscle caused an inflammatory response and insulin resistance, and fat tissue secreted adipokines and cytokines induced a decrease in skeletal muscle mass and function, leading to the development of sarcopenia (14–16). When insulin resistance occurred, the gluconeogenesis process in myocytes was promoted, resulting in decreased protein synthesis and increased catabolism, which lead to decreased muscle mass (17). Then, for lack of physical activity, it lead to abdominal fat deposition, systemic inflammatory response, and insulin resistance, further reducing the body’s lipid and glucose oxidation (18). Finally, some studies had found that patients with sarcopenia also have higher levels of inflammatory cytokines and inflammatory indicators that were involved in the activation of apoptosis, leading to a decrease in myofilament protein synthesis, which lead to sarcopenia (19). It shown in our study that preliminary confirmation was published about the relationship between sarcopenia and the prevalence of kidney stones. Sarcopenia might contribute to the development and progression of kidney stones through increasing obesity, insulin resistance, inflammation, and decreasing recreational activities.

In conclusion, we proved the associations between sarcopenia and the odds of kidney stones while controlling for potential variables, though several limitations still existed. First of all, we could not be completely convinced of the relationship between sarcopenia and kidney stones, because the study was a cross-sectional study. It needed more exploration to verify. Then, there was no consideration about relationship between position, type, and size of kidney stone and sarcopenia, and the basic characteristics of kidney stones was useful for prevention of kidney stones. Therefore, we should perform more studies to explore the potential mechanisms and to identify causality.

## Data availability statement

Publicly available datasets were analyzed in this study. These data can be found here: National Health and Nutrition Examination Survey (NHANES).

## Author contributions

BP, CX, and YW: conception and design. CT, HZ, and JN: administrative support. YW, YZ, and HS: provision of study materials or patients. TZ, YZ, and HZ: collection and assembly of data. CX, YZ, and HZ: data analysis and interpretation. All authors contributed to the article and approved the submitted version.

## Funding

This study was financially supported by the Shanghai Association for Science and Technology Commission (Grant No. 21142203400), National Natural Science Foundation of China (Grant No. 81870517 and 32070646), and the National Key Research and Development Program of China (2021YFC2009300 and 2021YFC200930X).

## Conflict of interest

The authors declare that the research was conducted in the absence of any commercial or financial relationships that could be construed as a potential conflict of interest.

## Publisher’s note

All claims expressed in this article are solely those of the authors and do not necessarily represent those of their affiliated organizations, or those of the publisher, the editors and the reviewers. Any product that may be evaluated in this article, or claim that may be made by its manufacturer, is not guaranteed or endorsed by the publisher.
