# Supplementary material for: Association between sarcopenia and kidney stones in United States adult population between 2011 and 2018
Source: Front Nutr. 2023 Mar 6;10:1123588. doi: 10.3389/fnut.2023.1123588 (PMC10025351; doi:10.3389/fnut.2023.1123588)
Supplement: Supplementary file 1 [file Table_1.docx]

**Table S1**. Baseline characteristics of participants between 2011 and 2018.

| **Characteristic** | **Total** | **No-sarcopenia** | **Sarcopenia** | **P**  **value** |
| --- | --- | --- | --- | --- |
|  | **No. (%)** | **No. (%)** | **No. (%)** |  |
| Total patients | 9472 | 8661 (91.4) | 811 (8.6) |  |
| Gender |  |  |  | 0.868 |
| Male | 4657 (49.2) | 4256 (49.1) | 401 (49.4) |  |
| Female | 4815 (50.8) | 4405 (50.9) | 410 (50.6) |  |
| Age |  |  |  | <0.001 |
| <39 years | 4793 (50.6) | 4507 (52.0) | 286 (35.3) |  |
| ≥40 years | 4679 (49.4) | 4154 (48.0) | 525 (64.7) |  |
| Race |  |  |  | <0.001 |
| Non-Hispanic white | 3428 (36.2) | 3204 (37.0) | 224 (27.6) |  |
| Non-Hispanic black | 1934 (20.4) | 1882 (21.7) | 52 (6.4) |  |
| Mexican American | 1374 (14.5) | 1100 (12.7) | 274 (33.8) |  |
| Other Hispanic | 948 (10.0) | 827 (9.5) | 121 (14.9) |  |
| Other | 1788 (18.9) | 1648 (19.0) | 140 (17.3) |  |
| Education level |  |  |  | <0.001 |
| Less than high school | 1610 (17.0) | 1366 (15.8) | 244 (30.1) |  |
| High school or equivalent | 2063 (21.8) | 1850 (21.4) | 213 (26.3) |  |
| College or above | 5799 (61.2) | 5445 (62.9) | 354 (43.6) |  |
| Marital status |  |  |  | 0.004 |
| Married | 4625 (48.8) | 4190 (48.4) | 435 (53.6) |  |
| Unmarried | 4847 (51.2) | 4471 (51.6) | 376 (46.4) |  |
| BMI (kg/m^2^) |  |  |  | <0.001 |
| <25.0 | 3001 (31.7) | 2932 (33.9) | 69 (8.6) |  |
| 25.0-29.9 | 2966 (31.3) | 2782 (32.1) | 184 (22.9) |  |
| ≥30.0 | 3497 (37.0) | 2947 (34.0) | 550 (68.5) |  |
| Hypertension |  |  |  | <0.001 |
| Yes | 2223 (23.5) | 1969 (22.7) | 254 (31.3) |  |
| No | 7249 (76.5) | 6692 (77.3) | 557 (68.7) |  |
| Smoking status |  |  |  | 0.001 |
| Never | 5733 (60.5) | 5218 (60.2) | 515 (63.5) |  |
| Former | 1613 (17.0) | 1457 (16.8) | 156 (19.2) |  |
| Current | 2126 (22.4) | 1986 (22.9) | 140 (17.3) |  |
| Alcohol use |  |  |  | <0.001 |
| Yes | 7040 (74.3) | 6506 (75.1) | 534 (65.8) |  |
| No/Unknown | 2432 (25.7) | 2155 (24.9) | 277 (34.2) |  |
| Vigorous recreational activities |  |  |  | <0.001 |
| Yes | 2967 (31.3) | 2844 (32.8) | 123 (15.2) |  |
| No | 6505 (68/7) | 5817 (67.2) | 688 (84.8) |  |
| Moderate recreational activities |  |  |  | <0.001 |
| Yes | 4277 (45.2) | 3997 (46.1) | 280 (34.5) |  |
| No | 5195 (54.8) | 4664 (53.9) | 531 (65.5) |  |
| Blood urea nitrogen (mg/dL) | 12.53±4.50 | 12.51±4.48 | 12.77±4.65 | 0.125 |
| Creatinine (mg/dL) | 0.85±0.37 | 0.86±0.36 | 0.78±0.40 | <0.001 |
| Uric acid (mg/dL) | 5.32±1.39 | 5.30±1.37 | 5.56±1.51 | <0.001 |

For categorical variables, P values were analyzed by chi-square tests. For continuous variables, the t-test was used.

Abbreviations: BMI, body mass index.
